# Systematic Review of l-Arginine for the Treatment of Hypoactive Sexual Desire Disorder and Related Conditions in Women

**DOI:** 10.3390/pharmacy9020071

**Published:** 2021-03-27

**Authors:** Nicole E. Cieri-Hutcherson, Andrea Jaenecke, Ajeet Bahia, Debra Lucas, Ann Oluloro, Lora Stimmel, Timothy C. Hutcherson

**Affiliations:** 1 Department of Pharmacy Practice, School of Pharmacy and Pharmaceutical Sciences, University at Buffalo, Buffalo, NY 14214, USA; 2 School of Pharmacy, D’Youville College, Buffalo, NY 14201, USA; a06jaenecke@hotmail.com (A.J.); ajeetbahia@gmail.com (A.B.); mcadal18@dyc.edu (L.S.); 3 Montante Family Library, D’Youville College, Buffalo, NY 14201, USA; lucasd@dyc.edu; 4 Department of Obstetrics and Gynecology, Jacobs School of Medicine and Biomedical Sciences, University at Buffalo, Buffalo, NY 14203, USA; aoluloro@buffalo.edu; 5 Department of Pharmacy Practice, School of Pharmacy, D’Youville College, Buffalo, NY 14201, USA; hutchert@dyc.edu

**Keywords:** arginine, hypoactive sexual desire disorder, sexual dysfunction, women’s health, dietary supplement

## Abstract

This systematic review evaluates the efficacy and safety of l-arginine alone or in combination for the treatment of women with hypoactive sexual desire disorder (HSDD) or related conditions, such as female sexual interest/arousal disorder and female sexual arousal disorder. Medline, Embase, International Pharmaceutical Abstracts, Science Direct, and the Cumulative Index to Nursing and Allied Health Literature were searched using keywords “arginine”, “Lady Prelox”, “ArginMax”, “Stronvivo”, “Ristela”, “hypoactive sexual desire disorder”, “female sexual interest arousal disorder”, “female sexual arousal disorder”, “sexual dysfunction”, “sexual behavior”, “dyspareunia”, “libido”, and permutations thereof. Relevant records were retained if they were primary literature, conducted in women with HSDD or related conditions, and published as full text in English. Five randomized controlled trials and two nonrandomized studies met eligibility criteria. Six of the seven studies reported either an increase in the total mean Female Sexual Function Index score or significant increases in multiple domains therein. One study assessed vaginal pulse amplitude and found a statistically significant increase in a combination treatment group compared to placebo. No significant side effects were reported. Four of seven studies had potential risk-of-bias concerns per Cochrane assessments. This systematic review found that combination products containing l-arginine in the form of ArginMax or Lady Prelox may be considered for the treatment of HSDD and related conditions in women regardless of age.

## 1. Introduction

Increasing awareness of female sexual dysfunction highlights a greater need for treatment strategies in women with hypoactive sexual desire disorder (HSDD) and related conditions. Medical definitions of sexual desire disorders have varied in the past and have undergone a significant revision from the Diagnostic and Statistical Manual of Mental Disorders, Fourth Edition, Revised (DSM-IV-TR) to the fifth edition (DSM V). Previously, DSM-IV-TR defined HSDD as persistent or recurrent deficient sexual thoughts and desire for sexual activity [1]. Additionally, female sexual arousal disorder (FSAD) was defined as persistent or recurrent inability to achieve and maintain adequate lubrication-swelling response of sexual excitement until completion of sexual activity. DSM V, combined HSDD and FSAD into the definition of female sexual interest/arousal disorder (FSIAD) [2]. FSIAD is defined as absence or significantly reduced sexual interest/arousal for at least 6 months. An absence/reduction of at least three of the following symptoms present: interest in sexual activity; sexual/erotic thoughts; initiation of sexual activity; responsiveness to partner’s attempt to initiate sexual activity; pleasure during sexual activity in at least 75% of encounters, interest/arousal in response to any internal/external cues, genital or non-genital sensations during sexual activity in at least 75% of sexual encounters. A provider may use the Decreased Sexual Desire Screener (DSDS) and a sexual history to confirm or diagnose HSDD [3]. 

A transition to FSIAD has caused debate amongst the scientific community and resulted in varying use of the aforementioned definitions. Some believe that validity and reliability of the diagnostic criteria remains to be established for FSIAD [4]. One study found that the majority of women with FSAD would not meet any of the six proposed criteria for FSIAD, even though they showed evidence of impairment relative to normal controls in each criteria [5]. Due to the controversy of FSIAD, as well as the distribution of data between three different definitions (primarily HSDD), evaluation of literature encompassing the three definitions is necessary for comprehensive understanding.

HSDD affects up to 8.9% of women in the United States (US) aged 18 to 44, 12.3% for those aged 45 to 64, and 7.4% for those older than 65 [6]. HSDD may lead to a decrease in perceived connection to sexual partner(s) or negative psychological and emotional states including impaired body image; decreased self-confidence and self-worth; and depression [3]. Patients with HSDD are more likely to experience memory problems, fatigue, back pain, depression, and an overall poor quality-of-life. As such, they utilize more healthcare services and have higher overall medical expenses due to an increased number of outpatient visits and use of prescription medications compared to patients without HSDD [7,8].

The exact etiology of HSDD and related conditions are unknown although it is likely multifactorial, including aspects related to biological, psychological, interpersonal, and sociocultural factors. Individual-specific factors also affect female human sexual desire such as intimacy, pleasure, partnership issues, sexual knowledge, and the wish to participate or behave in a sexual fashion. Acute and chronic stress, lack of sleep, mental and emotional health, and the well-being of both partners are additional factors that affect desire [9]. Postpartum states, lactation, menstrual cycle, hysterectomy, oophorectomy, menopause, stress, and medications such as hormonal contraceptives, psychotropic medications, and antihypertensive medications may impact sexual desire [9]. Sex steroids and neurotransmitters appear to play a key role [10]. Additionally, an imbalance between excitatory and inhibitory processes is thought to contribute to HSDD and related conditions. Oxytocin and norepinephrine modulate excitatory pathways that result in sexual arousal. Dopamine and small protein hormones modulate sexual desire in this pathway. The inhibitory pathway includes action from serotonin, opioids, and endocannabinoids which result in reduced sexual desire, regulation of sexual reward, and sedation. Nitric oxide (NO) may also affect sexual function by mediating vascular smooth muscle relaxation. Smooth muscle relaxation may then lead to increased lubrication and wall engorgement of the vagina, expansion of the vaginal luminal diameter, as well as menopause-associated vaginal atrophy. NO also influences clitoral cavernosa smooth muscle relaxation leading to an increase in artery inflow, increase in intra-cavernosal pressure, and clitoral engorgement [11]. 

The management of sexual dysfunction should be assessed using patient-reported outcomes to monitor treatment-associated changes [12]. Sexual disorders are most commonly measured using the Female Sexual Function Index (FSFI). The FSFI, though, is distinct from the DSDS, which is more commonly used to diagnose HSDD and related conditions. The FSFI is a self-administered tool that assesses physical and behavioral aspects of sexual function, although not psychological aspects [13]. The FSFI is a questionnaire assessing six separate domains of female sexual function (desire, arousal, lubrication, orgasm and satisfaction). There is a 19-item, 9-item, and 6-item version. Vaginal pulse amplitude (VPA) may also be used to assess female sexual function [14].

Flibanserin is the only oral FDA-approved medication for HSDD, specifically indicated for premenopausal women [3,6]. Flibanserin is a non-hormonal, serotonin 1A agonist and serotonin 2A antagonist. Flibanserin showed clinically and statistically significant improvement in the outcomes of physical and emotional distress, number of sexually satisfying events, and level of sexual desire compared to placebo [3]. The most common adverse effects include dizziness, somnolence, nausea, and fatigue, which may be mitigated via nighttime dosing. The use of flibanserin is restricted to a Risk Evaluation and Mitigation Strategy (REMS) program in order to increase awareness of the high risk of syncope and severe hypotension that occurs when taken with alcohol [15]. 

Other potential off-label prescription treatment options for HSDD and related conditions include bupropion, androgen therapy, sildenafil citrate, bremelanotide, and ospemifene. Vaginal moisturizers or lubricants, systemic estrogen, and local vaginal estrogens may also be used [6,16]. Complementary health approaches (CHA) have varying levels of clinical data. Common CHA products may include, either alone or in combination, ginkgo biloba, damiana leaf, ginseng, yohimbine, dehydroepiandrosterone (DHEA), l-arginine, and others [16]. 

l-arginine, either alone or in combination, has been used for HSDD and related conditions in women. It is a naturally occurring precursor to NO and a key mediator involved in circulation and sexual function [10]. l-arginine is converted to NO via nitric oxide synthase (NOS) causing an increase in NO and cyclic guanosine monophosphate (cGMP) which ultimately affects circulation and sexual function. l-citrulline, another naturally occurring amino acid, is converted in part to l-arginine in the body, raising the levels of l-arginine and increasing its effect on NO [17]. l-arginine and its effect on NO can lead to vasodilation and increase arterial blood flow to the genitals [18].

l-arginine is available as a combination product under the trade names ArginMax (The Daily Wellness Company; Honolulu, HI, USA), Lady Prelox (Pharma Nord Inc.; Hamilton, NJ, USA), Ristela (Bonafide Health, LLC; Harrison, NY, USA), and Stronvivo (Effective Delivery Systems, LLC; Houston, TX, USA). ArginMax combines ginkgo biloba, damiana leaf, Korean ginseng, and l-arginine, along with a variety of vitamins and minerals [10]. Lady Prelox is a combination product composed of pine bark extract, l-arginine, l-citrulline, and rose hip extract [13,19,20,21]. Ristela is composed of French Maritime Pine Bark extract, l-arginine, l-citrulline, and rose hip extract [22]. Stronvivo is composed of l-arginine, l-citrulline, l-carnitine, zinc, and magnesium. Ginseng’s primary active components are ginsenosides, which have been shown to increase NO production in endothelial cells. Ginkgo biloba effects microvascular circulation through the action of smooth muscle relaxation via the NO pathway. Damiana leaf is thought to have receptor activity as a phyto-progestin [10]. French Maritime pine bark extract may help improve endothelial function and increase vasodilation. Mechanistically, rose hip extract is not directly related to NO or sexual function, but rather has an antioxidative effect [13,20,21,23]. While additional products exist, including products in combination, ArginMax and Lady Prelox are the only two proprietary blends with published literature at this time.

These products have been tested and used for male sexual dysfunction with some degree of success [24]. It is hypothesized that a similar mechanism of action in women may cause an increase in sexual arousal similar to that experienced by men. This systematic review evaluates the efficacy and safety of l-arginine, as monotherapy and in combination products, for the treatment of women with HSDD or related conditions. 

## 2. Materials and Methods 

### 2.1. Protocol and Registration

Study reporting followed Preferred Reporting Items for Systematic Reviews and Meta-Analyses standards [25]. A protocol was not registered with a third party. Neither the study nor its investigators received external funding. The authors have no financial conflicts of interest to disclose regarding the development or dissemination of this manuscript.

### 2.2. Information Sources and Search Strategy

Searches of Medline, Embase, and International Pharmaceutical Abstracts (IPA) sought records regarding the administration of l-arginine, either alone or in combination with other products, in women with HSDD for improved sexual desire and related outcomes. Confirmatory searches of Science Direct and the Cumulative Index to Nursing and Allied Health Literature (CINAHL) were conducted separately to verify rigor of the search strategy and therefore, were not included in the record count. Database searches were conducted spanning origin to 11 November 2020. Study authors were not contacted for additional data. Automated search limits were not applied during the conduct of the searches. A Medline-based search strategy is provided below; equivalent searches were used for other databases listed.

(arginine or lady prelox or ArginMax or Stronvivo or Ristela) and
(hypoactive sexual desire disorder or female sexual interest arousal disorder
or female sexual arousal disorder or (exp sexual dysfunctions, psychological)
or sexual dysfunction or sexual behavior or dyspareunia or libido)

### 2.3. Study Selection

Resultant records were screened by title and abstract, followed by a full-text review of the remaining records. Study selection was performed in duplicate by two members of the investigative team. Discordant records were reconciled by consensus of three or more members of the investigative team. The following eligibility criteria were used to identify records that were accessible and applicable to the study objective: conducted in humans; conducted in women with HSDD or related conditions, either in total or in part per subgroup analysis; reported on any relevant efficacy or safety outcomes of HSDD or related conditions; available in English; and identified as published primary literature. Ineligible literature types were those that did not have a corresponding published manuscript, such as abstract-only records, professional posters, conference proceedings, or grey literature. Bibliographic review of eligible records was conducted to confirm the rigor of the search strategy and identify other relevant records, which were subsequently subjected to the same screening process described above.

### 2.4. Data Collection

Data was extracted from eligible records using standardized forms and included the following items: study identifiers; data necessary for risk-of-bias assessments; study design and key methodological characteristics; intervention and comparators or controls, if applicable; all reported efficacy or safety outcomes of HSDD or related conditions; and relevant participant demographics. Data extraction and verification were performed in duplicate by two members of the investigative team. Discordant data found after extraction was reconciled by consensus of three or more members of the investigative team.

### 2.5. Risk of Bias Analysis

Eligible studies were assessed for risk-of-bias using the revised Cochrane risk-of-bias tool for randomized studies (RoB2 and RoB2 for Crossover trials) and risk of bias in non-randomized studies of interventions (ROBINS-I) tool [26,27,28]. Risk-of-bias assessments were conducted at the study level and reported using the outcome measures and study characteristics, as described above. A risk-of-bias assessment was then tabulated across the resultant literature.

## 3. Results

**Figure 1:**
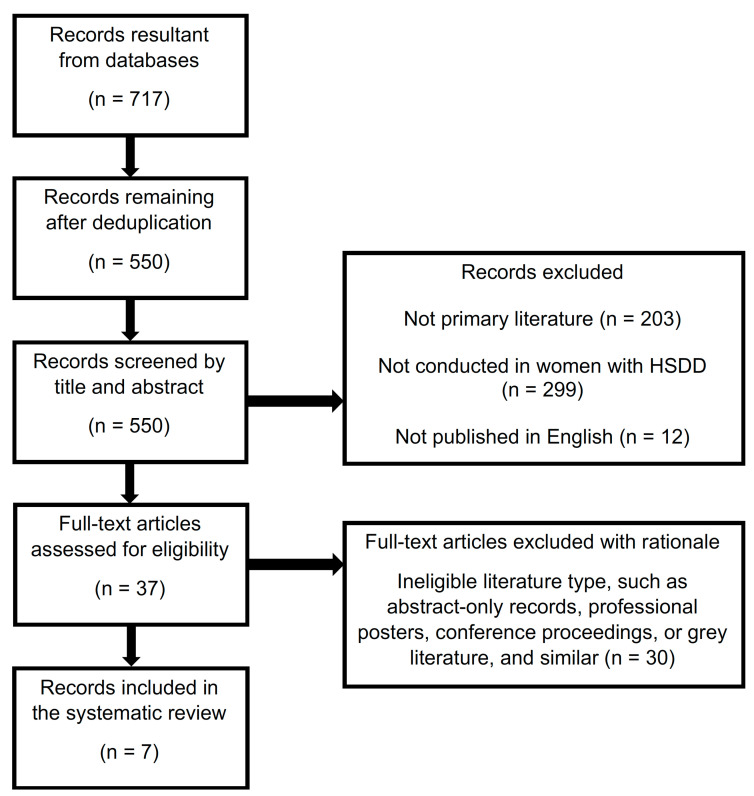
Screening Process—Inclusion and Exclusion Criteria.

The initial search of Medline, Embase, and IPA resulted in 717 total records, with 550 following deduplication. No additional records were captured from searches of Science Direct and the CINAHL. After initial screening 37 potential records remained, seven of which met eligibility criteria [10,13,19,20,21,29,30]. The screening process after application of eligibility criteria may be seen in Figure 1. Resultant records included five randomized, placebo-controlled trials and two nonrandomized pilot studies that assessed the effect of l-arginine alone or in combination with other supplements in women with HSDD or related conditions. Investigational products included l-arginine in combination with yohimbine; l-arginine in combination with Korean ginseng root extract, ginkgo biloba leaf extract, and damiana, along with a variety of vitamins and minerals (ArginMax); or l-arginine in combination with l-citrulline, rose hip extract, and pycnogenol (Lady Prelox). Study tools and outcomes included: FSFI; VPA; oxidative stress; subjective ratings of arousal; menopausal symptoms scores; presence of vaginal mucus; and other adverse events associated with sexual dysfunction.

Ito et al. (2001) conducted a double-blinded, placebo-controlled, superiority study that evaluated the role of ArginMax for the improvement of female sexual dysfunction [10]. Participants were included in this 4-week study if they were at least 21 years of age and wanted to improve their sexual function. Participants were either given ArginMax or a matching placebo and the specific dosing regimen was not discussed. The primary outcome measure was the FSFI and the mean improvement from baseline to four weeks was measured. Patient demographics may be seen in Table 1. FSFI outcome measure results may be seen in Table 2. Additional outcomes and safety data may be found in Table 3. None of the participants reported any significant side effects. Overall, there were notable improvements that were seen in the overall sex life in the treatment group compared to the control group (*p* < 0.01). Limitations of this study included a small sample size, missing or poorly defined exclusion criteria, and concurrent drug use was not discussed. 

Meston et al. (2002) conducted a randomized, double-blinded, placebo-controlled, single centered, superiority study that assessed the effects of yohimbine in combination with l-arginine compared to yohimbine alone and a placebo group on subjective ratings of sexual arousal and physiological sexual arousal for female sexual arousal disorder in postmenopausal women [29]. Women 21 years or older who met the DSM-IV criteria for acquired, generalized, FSIAD; postmenopausal for at least six months, and were experiencing trouble attaining or maintaining lubrication during sexual activity over at least the past six months were included. If the subject was using antidepressants, clonidine, alpha blockers, if they had a medical disorder that could affect sexual functioning or participated in another investigational study in the past 30 days, the subject was excluded. Participants had to complete four visits: one baseline and three to compare the treatment arms, over the course of five weeks. At each visit the subjects were either given three 2 mg tablets of yohimbine and 6 g of l-arginine; three 2 mg tablets of yohimbine and l-arginine placebo; or identical placebo replacements of both. The primary outcome of this study was VPA response with a secondary outcome measure of self-reported ratings of sexual arousal. Patient demographics may be seen in Table 1. VPA, additional outcomes and safety data may be found in Table 3. The study did not report any serious adverse events that occurred during treatment. The authors concluded that the addition of l-arginine’s mechanism of action to yohimbine’s mechanism is a safe and effective way to enhance genital vasocongestion in postmenopausal women who have female sexual arousal disorder; however, authors did state that it would help a woman’s subjective ratings of sexual arousal. Limitations of this study included a small sample size, environmental impact on women’s subjective measures of sexual arousal, and the exclusion of an individual l-arginine treatment arm. 

Ito et al. (2006) conducted a placebo-controlled, double-blinded, randomized, superiority study that assessed the role of dietary supplementation with ArginMax on sexual function in premenopausal, perimenopausal, and postmenopausal women [30]. Subjects that were included were recruited via local advertisements and expressed interest in improving their current sexual function. Participants were included in this study if they had trouble becoming aroused or if they suffered a lack of sexual desire. Participants were given either ArginMax or a matching placebo; the dosing regimen of the ArginMax was not disclosed. Participants were allowed to continue taking medications that they were using prior to enrollment in this study. There was an even distribution between groups for participants that were previously treated for depression, hypertension, and sexual dysfunction, including previous use of testosterone, counseling, traditional hormonal therapy. The primary outcome measure was the FSFI at baseline and at four weeks. Patient demographics may be seen in Table 1. FSFI outcome measure results may be seen in Table 2. Additional outcomes and safety data may be found in Table 3. Four participants reported minor gastric disturbances, two reported heavier menstrual bleeding, and one reported increased incidence of headache. No other significant side effects were reported. Ito et al. (2006) concluded that intervention with ArginMax had a positive effect on sexual desire scores among pre, peri, and postmenopausal women. Limitations of this study included a small sample size, missing or poorly defined exclusion criteria, concurrent drug use was not discussed, and the statistical test was inappropriate for the type of data that was collected.

Bottari et al. (2012) conducted a randomized, single-blinded, placebo-controlled, superiority study that evaluated the efficacy of Lady Prelox for improving and controlling mild to moderate sexual dysfunction in generally healthy postmenopausal women [20]. Participants were included in this study if they were healthy, postmenopausal women aged 45 to 55 years old with no clinically significant cardiovascular disease and moderate sexual dysfunction. Participants had normal thyroid function and metabolic parameters, no surgery or hormonal treatment within the past 12 months, and a BMI <24 kg/m^2^. Investigators required a satisfactory education level and cultural-social status were considered important for inclusion for understanding and adherence to the study protocol. Specific exclusion criteria were not listed. Participants were given two tablets of Lady Prelox in the morning and 2 tablets in the evening or a matching placebo over the course of 8 weeks. The primary objective was improvement in FSFI. Each group required 20 subjects to complete the study period in order to meet power; 36 participants completed the study period in the treatment group and 39 completed the study in the control group. Results were reported as total means, and were collected at baseline, four weeks and eight weeks. In the treatment group, the mean scores and standard deviations were reported. Patient demographics may be seen in Table 1. FSFI outcome measure results may be seen in Table 2. Additional outcomes and safety data may be found in Table 3. No significant side effects were reported. The authors concluded that women supplemented with Lady Prelox significantly improved in all six domains of FSFI over four weeks, with limited improvement over the last four weeks. Limitations of this study included a small sample size. 

Bottari et al. (2013) conducted an independent, nonrandomized, single-blinded, pilot registry study that assessed the efficacy of Lady Prelox in sexual dysfunction in healthy women of late reproductive age (defined as around 40 years of age) [13]. Participants were not randomized between groups and were controlled by a best management group. Participants were included if they were women who successfully filled out the FSFI questionnaire and whose score was between 15 and 24. Women had to be in a stable monogamous relationship with a male partner for at least six months prior to follow up period and were not older than 45 years of age with regular menstrual periods for prior six months. Authors required a high school education, stable job, and permanent address as important for inclusion in this study in order to ensure understanding of the study protocol. Women with a baseline oxidative stress measurement of >350 Carr Units were included. Women were excluded from the screening process if there was a possible clinical cardiovascular disease or significant metabolic or cardiovascular risk factors needing treatment and from the follow up period if the women had a psychiatric disorder, addiction, or history of drug or alcohol abuse. Patients were given Lady Prelox over the course of eight weeks. The primary objective was the FSFI, with a secondary outcome of plasma free radicals (PFR). Patient demographics may be seen in Table 1. FSFI outcome measure results may be seen in Table 2. Additional outcomes and safety data may be found in Table 3. There were no side effects reported. The authors concluded that supplementation with Lady Prelox significantly improves sexual function across all domains of FSFI in healthy women and is associated with a significant decrease in oxidative stress. Limitations of this study included a small sample size, insufficient randomization, and blinding.

Stanislavov R. et al. (2014) conducted a randomized, double-blinded, placebo-controlled, superiority study that evaluated the efficacy of Lady Prelox for improving and controlling moderate sexual dysfunction in otherwise healthy peri-menopausal women [19]. Participants were included in this study if they were women aged 40 to 50 years old with moderate sexual dysfunction and a stable monogamous relationship for more than six months. Stable socio-economic living circumstances were also required for inclusion, although specifics as to these factors were not mentioned. Exclusion criteria included hormone replacement therapy, contraceptive medication, oophorectomy, hysterectomy, cardiovascular disease, renal disease, liver disease, hypertension, diabetes mellitus, psychiatric disorders, or concomitant treatment of sexual dysfunction. Prior treatment of sexual dysfunction or a required wash-out period for participants who recently received a prior study medication were not discussed. Participants were given two tablets of Lady Prelox in the morning and two tablets in the evening or a matching placebo over the course of eight weeks. The primary outcome measure was FSFI. Results were reported as total means and were collected at baseline, four weeks, and eight weeks. In the treatment group, the mean scores and standard deviations (SD) were reported. Patient demographics may be seen in Table 1. FSFI outcome measure results may be seen in Table 2. Additional outcomes and safety data may be found in Table 3. No significant side effects reported. The authors concluded that women supplemented with Lady Prelox significantly improved in all six domains of FSFI over eight weeks. Limitations of this article included a small sample size and differences between groups at baseline in favor of Lady Prelox. 

Cesarone et al. (2019) conducted a non-randomized pilot registry study that compared Lady Prelox to standard management in the improvement of signs and symptoms associated with vaginal dryness in premenopausal and postmenopausal women [21]. This study was neither randomized nor blinded and was controlled by a group of standard management participants not offered Lady Prelox. Participants included in this study were women with moderate sexual dysfunction that began in the past three years with normal thyroid function and other metabolic parameters, normal estrogen levels, a regular menstrual cycle, minimum of one child before evaluation (latest child at least five years before inclusion) and normal pregnancies, a BMI < 26 kg/m^2^ with good educational, social and professional life status to help with their understanding of the study protocol and no clinical or risk conditions present. Premenopausal women between the ages of 40–50 and postmenopausal women between the ages of 50–60 were included. Specific exclusion criteria were not listed. Eligible participants were given Lady Prelox two tablets in the morning and the evening for eight weeks. The primary objective of this study was the FSFI with a focus on vaginal dryness and a secondary objective of the menopause symptom score questionnaire. The groups were similar in both pre and postmenopausal groups. Patient demographics may be seen in Table 1. FSFI outcome measure results may be seen in Table 2. Additional outcomes and safety data may be found in Table 3. There were no side effects or tolerability issues noted. The authors concluded that the effects of supplementation with Lady Prelox on vaginal dryness and related symptoms appear to be significant and rapidly achieved. Limitations of this study included a small sample size, lack of randomization, and insufficient blinding.

The following limitations were observed across the eligible records: concurrent drug use not discussed (*n* = 2); missing or poorly defined exclusion criteria (*n* = 2); environmental impact on outcome measures not taken into consideration (*n* = 1); differences between groups at baseline (*n* = 1) insufficient randomization and blinding (*n* = 2); and a small sample size (*n* = 7). Risk-of-bias assessments were conducted for each of the seven records using the appropriate tool for each study design; results may be seen in Table 4. The randomized controlled trials by Ito et al. (2001), Ito et al. (2006), Bottari et al. (2012), and Stanislovov et al. (2014) were assessed using the RoB2 tool [10,19,20,28,30]. Both studies conducted by Ito et al. (2001, 2006) had low risk-of-bias in all domains and for the study overall [10,30]. Bottari et al. (2012) was a single blinded study where the investigators were aware of the treatment arms [20]. This may have influenced the results leading to potential concerns for risk of bias. All the other domains were considered low risk-of-bias, so the overall study had the potential for some concerns. Stanislovov et al. (2014) had low risk of bias in all domains except for randomization process due to differences between groups at baseline, leading potential concerns in that domain and overall [19]. In addition, the two nonrandomized controlled studies were analyzed via the ROBINS-I tool [27]. Bottari et al. (2013) and Cesarone et al. (2019) were both open label, which may have influenced the results reported [13,21]. Potential risk of bias may have existed due to confounding effects of co-interventions. Additionally, Bottari et al. (2013) had 10 dropouts that were re-evaluated outside of the planned observation period and as such they were not included in the final analysis [13]. Both studies had potential for serious risk-of-bias due to these factors [13,21]. 

## 4. Discussion

The available data suggests that pre-, peri-, and post-menopausal women suffering from HSDD and related conditions may benefit from the use of a product containing l-arginine. The included studies were a maximum of two months in duration, limiting both the potential observation of efficacy and, more importantly, longer-term safety outcomes. Significant variability may exist during the peri-menopausal period as it relates to cycle length and the impact that this may have on response. It remains to be seen whether significant effects observed in these studies would continue in women as they transition from peri- to post-menopausal status, although significant data in both populations was reported. Again, this may be confounded by the short duration of the studies included. Known risks to any one of the multiple ingredients in the combination products reviewed may be exclusions for use in terms of adverse effects, relevant co-morbidities, and concomitant medications. Although not reported in the studies, caution should be considered in the use of products also containing Korean ginseng due to the potential for vaginal bleeding and the need to assess these patients for endometrial cancer [31].

Overall, six of the seven studies reviewed used the FSFI to measure sexual arousal in participants [13,19,20,21]. Two studies assessing oxidative stress reported statistically significant decreases from baseline [13,21]. One study assessing VPA showed a significant change in the l-arginine and yohimbine combination compared to baseline [29]. None of the seven studies reported any significant side effects; the most common were minor gastric occurrences, heavier bleeding during menstrual cycle, and headache. Doses of l-arginine up to 30 g daily had been shown previously to be safe when taken daily and are well tolerated, but may cause gastric disturbances such as abdominal pain, nausea and diarrhea, insomnia, and headaches in adults [32]. 

There is no data demonstrating the effectiveness of l-arginine monotherapy (non-combination) in the treatment of HSDD or related conditions. All seven records included in this review assessed l-arginine combination products, and it is thus difficult to establish a positive correlation between l-arginine monotherapy and effect. The normal daily regimen of Lady Prelox is two tablets each morning and evening, with each tablet containing 200 mg of l-arginine, equal to 800 mg per day [20]. The ArginMax regimen is three capsules each morning and evening, with each capsule containing 348 mg of l-arginine per capsule, equal to 2088 mg of l-arginine per day [33]. The use of these products has been studied for up to eight weeks, with the greatest effect being seen after four weeks. If the patient does not experience benefit in their feelings of desire, lubrication, arousal, orgasm, satisfaction, or pain in that time, use of these products should be discontinued. CHAs commonly have a lack of data surrounding the efficacy and safety of use.

ArginMax proprietary blend consists of 77% l-arginine and 23% mixture of Korean ginseng, ginkgo biloba, and damiana leaf, with a total amount of the proprietary blend for six capsules of 2700 mg [33]. As both ginkgo biloba and Korean ginseng are thought to have some effect on NO, similar to l-arginine, this may provide a synergistic effect. Given that 23% of ArginMax is a proprietary blend and does not have a dose defined per active ingredient, the maximum potential daily doses of Korean ginseng, ginkgo biloba, and damiana leaf, could be up to 612 mg for any one component. Potentially effective daily dose ranges of these ingredients for HSDD or related conditions per available literature are 120 mg to 300 mg for ginkgo biloba, 2000 to 4000 mg for damiana leaf, and 3000 mg for Korean ginseng [34,35,36]. Korean ginseng and damiana at the maximum potential daily doses in ArginMax are below those daily doses studied in persons for HSDD or similar indications. Ginkgo biloba at the maximum daily dose in ArginMax would be above the minimum daily dose studied in persons for HSDD or similar indications. A placebo-controlled, triple-blinded, randomized superiority study was done to test the effects of ginkgo biloba 240 mg daily to males and females with antidepressant-induced sexual impairment that found that there were no statistically significant differences between the placebo and treatment groups [37]. This is suggestive of l-arginine being either the key active ingredient in the combination or a major contributor to the therapeutic effect if the combination does, in fact, rely on a synergistic mechanism; hence, there is a need for further studies in this patient population using l-arginine alone. Of note, Korean ginseng (present in ArginMax) has the potential to cause vaginal bleeding. This may be a concern as was discussed previously [32].

Alternatively, Lady Prelox does not contain Korean ginseng. Lady Prelox contains 200 mg of both l-arginine and l-citrulline along with 20 mg of French Maritime pine bark extract and 50 mg of rose hip extract [20]. French Maritime pine bark extract may help improve endothelial function and increase vasodilation and has been studied in the use of sexual dysfunction in doses of 20–50 mg daily [13,20,21,23]. As all components of Lady Prelox are in this supplement at the same dosage, it is difficult to determine how much of the effect is due to the l-arginine itself, or the additive effects of l-citrulline. None of the dosages in any of these products would be large enough to cause significant side effects [38,39,40]. Again, further studies are warranted to clarify the effects seen in studies included in this systematic review.

The instruments used during the studies have inherent limitations. The FSFI is the most widely used and validated tool to assess sexual dysfunction. It does not, however, assess psychological aspects that may impact HSDD and related conditions. Additionally, social and cultural factors that may impact sexual function are not assessed by the FSFI. Diagnosis via FSFI or alternative methods for inclusion in studies was often absent. All but one study used the FSFI to assess improvements in sexual function. The VPA is not without limitation, as well, as it assesses physiological measurements of sexual function and not behavioral or psychological aspects. The VPA is measured in a lab setting which may affect the study participants’ ability to feel sexual arousal compared to settings in which sexual activity may more commonly occur. Oxidative stress was measured in two of the seven studies [13,21]. The use of either the FSFI or VPA as measurement in the studies included were considered strengths, however, none of the studies used both measurements which could be considered a limitation.

In comparing l-arginine containing products to flibanserin, the only oral FDA-approved medication for the management of HSDD, adverse effects, population of study, and cost may be a consideration. Flibanserin carries a risk of hypotension, somnolence, syncope, fatigue, and carcinogenicity, and a REMS program has been enacted to ensure the safe use of flibanserin [41,42]. By contrast, limited adverse effects have been reported with l-arginine. Additionally, flibanserin is only approved for premenopausal women, while l-arginine has been studied in pre- and post-menopausal women. A daily cash price for flibanserin is $20.90 (100 mg tablet daily), whereas ArginMax is approximately $1.07 per daily serving and Lady Prelox is approximately $4.35 per daily serving (as per trending costs across multiple online vendors) [33,43]. 

Considering the current paucity of robust data in this population, additional studies would be valuable. Although it has been established that sexual dysfunction is more common in women compared to men, a disparity exists in the relative degree of research conducted in this area. Multiple studies have been conducted assessing the effect of l-arginine alone or in combination in men with erectile dysfunction. Conversely, a paucity of data exists for the use of l-arginine in women reporting sexual dysfunction. Increasing awareness of female sexual dysfunction highlights a greater need for treatment strategies in women with HSDD or related conditions. Due to the lack of data regarding a specific biological mechanism explaining female sexual dysfunction, it is difficult to determine a pharmacologic target, which may account for the lack in pharmacotherapeutic options for HSDD and related conditions. Most medications used in this setting aim to address an underlying factor such as mental health disorders or hormonal imbalances. Race may influence the experience of sexual dysfunction, including HSDD. In a national survey, Hispanic women reported fewer sexual problems compared to white and black respondents. Black women more often reported low sexual desire, and white women reported higher levels of sexual pain [44]. Unfortunately, race was only reported in one of the records included in this study, limiting external validity based on race. Of participants in Meston et al., 88% were Caucasian [29]. Additionally, three of the seven studies explicitly stated participants had to be in good, social, educational or professional standing to be included in the study [13,19,20]. The eligibility criteria of these studies were vague, are of relative value depending upon the audience, and may limit reproducibility and external validity of the results.

Limitations of this systematic review include a lack of well-powered randomized controlled trials. There was also a lack of studies that assessed l-arginine alone in the treatment of women with HSDD or related conditions. Conference posters were excluded from this study to avoid duplication of results. Studies evaluating l-arginine for cancer management were excluded due to potential for confounding factors influencing low sexual desire in this population. There are currently no clinical trials in progress assessing the effect of l-arginine alone in male or female participants related to sexual dysfunction. Investigators identified three completed studies of l-arginine for erectile dysfunction male participants [45].

All literature that met eligibility criteria have important potential risk-of-bias concerns. All seven studies included in this systematic review had small sample sizes and 57% of the studies (four of seven) also had additional concerns related to risk-of-bias. Both Ito et al. studies (2001 and 2006) had missing or poorly defined exclusion criteria [10,30]. Stanislovov et al. (2014) had differences between groups at baseline in favor of the investigational medication [19]. All studies administered l-arginine as part of a combination therapy, making it difficult to attribute significant effects to l-arginine alone or as part of a combination product [10,13,19,20,21,29,30].

Based on the analysis of the active ingredients in ArginMax and Lady Prelox, it may suggest that l-arginine is a major contributor to the effects seen in the included studies, along with l-citrulline in the case of Lady Prelox. This conclusion is not evidence-based and as such additional research is warranted to assess the effect of l-arginine alone on women with HSDD or related conditions. Ideally, these studies would be well-powered, double-blinded, randomized controlled trials with both placebo and active comparators against l-arginine monotherapy.

## 5. Conclusions

Based on the results from this systematic review, l-arginine, as part of a combination product, may be considered for the treatment of HSDD in women, regardless of age. There is a lack of high-quality evidence supporting the use of l-arginine alone, and it is difficult to extrapolate the benefits of combination products to l-arginine specifically. Additional research is warranted to determine the efficacy of l-arginine monotherapy in women with HSDD.

## Figures and Tables

**Table 1:** Patient Demographics [10,13,19,20,21,29,30].

	*n*	Age (years)	Height (Mean, cm)	Weight (Mean, kg)	Ethnicity	Concurrent Use of Hormonal Therapy	Reported Sexual Dysfunction	Previous Treatment for Sexual Dysfunction
Ito et al. RCT (2001)								
	77	22–71	NR	NR	NR	NR	NR	6
Meston et al. RCT (2002)								
	24	27–69	159.5	70.7	Caucasian (88%) Hispanic (4%) African American (4%) Asian (4%)	NR	NR	NR
Ito et al. RCT (2006)								
Total	108	22–73	NR	NR	NR	ArginMax (*n* = 18) Placebo (*n* = 16)	NR	*n* = 12
Pre-menopausal	59	22–48	NR	NR	NR	NR	NR	*n* = 12
Perimenopausal	20	36–57	NR	NR	NR	3	NR	NR
Postmenopausal	29	42–73	NR	NR	NR	18	NR	NR
Bottari et al. RCT (2012)								
	80	45–55	NR	NR	NR	Exclusion criteria	Moderate sexual dysfunction	NR
Bottari et al. NRCT (2013)								
	100	37–45	NR	NR	NR	All women reported taking oral contraceptives	NR	NR
Stanislavov et al. RCT (2014)								
	80	40–50	NR	NR	NR	Exclusion criteria	Moderate sexual dysfunction	NR
Cesarone et al. NRCT study (2019)								
Pre-menopausal	67	40–50	NR	NR	NR	Exclusion criteria	Moderate sexual dysfunction	NR
Post-menopausal women	73	50–60	NR	NR	NR	Exclusion criteria	Moderate sexual dysfunction	NR

Abbreviations: NR: not reported; NRCT: nonrandomized controlled trial; RCT: randomized controlled trial.

**Table 2:** FSFI Outcome [10,13,19,20,21,29,30].

	*n*	Mean Total Scores	Desire ^ab^	Sexual Arousal	Lubrication ^1^	Orgasm	Satisfaction ^cd^	Discomfort and Pain	Frequency of Intercourse	Degree of Clitoral Sensation
Ito et al. RCT (2001) ^4^										
ArginMax	34	NR	70.6% ^a^ 61.8% ^b^	NR	52.9%	47.1%	73.5% ^c^ 61.8% ^d^	38.2%	64.7%	52.9%
Placebo	43	NR	41.9% ^a^ 34.9% ^b^	NR	25.6%	30.2%	37.2% ^c^ 34.9% ^d^	18.6%	25.6%	34.9%
			*p* < 0.01 ^a^ *p* = NR ^b^	NR	*p* < 0.01	*p* < 0.07	*p* < 0.01 ^c^ *p* < 0.01 ^d^	*p* < 0.03	*p* < 0.01	*p* < 0.06
Ito et al. RCT (2006) ^3^										
ArginMax Premenopausal	25	NR	72% ^a^ 60% ^b^	NR	40%	44%	68% ^c^ 52% ^d^	32%	56%	52%
Placebo Premenopausal	34	NR	47% ^a^ 38% ^b^	NR	29%	29%	35% ^c^ 35% ^d^	24%	26%	38%
			*p* = 0.03 ^a^ *p* = 0.05 ^b^	NR	*p* = 0.20	*p* = 0.13	*p* = 0.007 ^c^ *p* = 0.10 ^d^	*p* = 0.23	*p* = 0.01	*p* = 0.15
ArginMax Perimenopausal	14	NR	57% ^a^ 57% ^b^	NR	64%	50%	71% ^c^ 79% ^d^	43%	86%	71%
Placebo Perimenopausal	6	NR	50% ^a^ 33% ^b^	NR	17%	17%	33% ^c^ 33% ^d^	17%	17%	33%
			*p* = 0.38 ^a^ *p* = 0.18 ^b^	NR	*p* = 0.03	*p* = 0.10	*p* = 0.06 ^c^ *p* = 0.03 ^d^	*p* = 0.16	*p* = 0.002	*p* = 0.06
ArginMax Postmenopausal	16	NR	50% ^a^ 31% ^b^	NR	25%	31%	44% ^c^ 50% ^d^	19%	38%	38%
Placebo Postmenopausal	13	NR	8% ^a^ 23% ^b^	NR	15%	31%	31% ^c^ 31% ^d^	15%	31%	15%
			*p* = 0.008 ^a^ *p* = 0.33 ^b^	NR	*p* = 0.29	*p* = 0.50	*p* = 0.25 ^c^ *p* = 0.16 ^d^	*p* = 0.43	*p* = 0.36	*p* = 0.11
Bottari et al. RCT (2012)										
LP Baseline	40	44.6 ± 24.1	5.13 ± 2.3 ^a^	9.2 ± 4.5	11.4 ± 5.8	6.5 ± 4.1	6.1 ± 3.9 ^c^	6.3 ± 3.5	NR	NR
LP 4 weeks	40	70.9 ± 18.5 (*p* < 0.05)	6.6 ± 2.1 ^a^	14.0 ± 4.5	16.0 ± 4.3	11.3 ± 2.9	11.3 ± 2.9 ^c^	11.7 ± 1.7	NR	NR
LP 8 weeks	40	71.7 ± 23.9 (*p* < 0.05)	7.0 ± 2.4 ^a^	14.5 ± 4.5	16.6 ± 5.2	11.1 ± 3.5	11.1 ± 3.5 ^c^	11.4 ± 3.7	NR	NR
Placebo Baseline	40	44.1 ± 22.8	5.2 ± 2.4 ^a^	9.2 ± 4.9	10.9 ± 5.1	6.4 ± 3.6	6.0 ± 3.4 ^c^	6.4 ± 3.4	NR	NR
Placebo 4 weeks	40	45.0 ± 21.4	5.0 ± 2.4 ^a^	9.3 ± 4.5	11.2 ± 4.5	6.7± 4.0	6.0 ± 3.7 ^c^	6.8 ± 3.3	NR	NR
Placebo 8 weeks	40	47.4 ± 21.8	5.7 ± 2.4 ^a^	11.3 ± 4.7	10.7 ± 4.4	6.6 ± 3.6	6.0 ± 3.7 ^c^	7.1 ± 3.4	NR	NR
Bottari et al. NRCT (2013) ^2^										
LP Baseline	49	14.96 ± 2.68	2.0 ^a^ 2.0 ^b^	NR	1.0	2.0	2.0 ^c^ 1.0 ^d^	1.0	1.0	2.0
LP 4 weeks	49	28.26 ± 2.35 (*p* = NR)	3.0 ^a^ 3.0 ^b^	NR	3.0	3.0	3.0 ^c^ 3.0 ^d^	3.0	3.0	3.0
LP 8 weeks	49	33.91 ± 2.7 (*p* < 0.001)	4.0 ^a^ 4.0 ^b^	NR	4.0	4.0	4.0 ^c^ 4.0 ^d^	4.0	4.0	4.0
BM Baseline	51	17.92 ± 2.32	2.0 ^a^ 3.0 ^b^	NR	1.0	2.0	2.0 ^c^ 2.0 ^d^	2.0	1.0	2.0
BM 4 weeks	51	23.45 ± 1.82	3.0 ^a^ 4.0 ^b^	NR	2.0	3.0	2.0 ^c^ 3.0 ^d^	2.0	2.0	2.0
BM 8 weeks	51	23.52 ± 2.2	3.0 ^a^ 3.5 ^b^	NR	3.0	3.0	2.0 ^c^ 3.0 ^d^	2.0	2.0	2.0
Stanislavov et al. RCT (2014)										
LP Baseline	40	16.50 (SD 2.85)	2.58 (SD 0.43)	2.85 (SD 0.59)	2.85 (SD 0.59)	2.85 (SD 0.59)	2.85 (SD 0.59)	2.52 (SD 0.53)	NR	NR
LP 1 month	40	21.65 (SD 2.79) (*p* < 0.001)	3.50 (SD 0.54) 58% (*p* < 0.001)	3.72 (SD 0.53) 62% (*p* < 0.001)	3.72 (SD 0.53) 62% (*p* < 0.001)	3.69 (SD 0.57) 62% (*p* < 0.001)	3.63 (SD 0.64) 61% (*p* < 0.001)	3.39 (SD 0.54) 57% (*p* < 0.001)	NR	NR
LP 2 months	40	26.49 (SD 3.28) (*p* < 0.001)	4.23 (SD 0.66) 71% (*p* < 0.001)	4.47 (SD 0.62) 75% (*p* < 0.001)	4.53 (SD 0.62) 76% (*p* < 0.001)	4.46 (SD 0.64) 74% (*p* < 0.001)	4.47 (SD 0.61) 75% (*p* < 0.001)	4.32 (SD 0.65) 72% (*p* < 0.001)	NR	NR
Placebo Baseline	40	12.42 (SD 2.25)	2.13 (SD 0.51)	2.13 (SD 0.51)	2.13 (SD 0.51)	2.13 (SD 0.51)	2.13 (SD 0.51)	1.77 (SD 0.61)	NR	NR
Placebo 1 month	40	14.40 (SD 0.82)	2.13 (SD 0.51) 36%	2.40 (SD 0.00) 40%	2.40 (SD 0.00) 40%	2.40 (SD 0.00) 40%	2.40 (SD 0.00) 40%	2.67 (SD 0.51) 45%	NR	NR
Placebo 2 months	40	16.62 (SD 2.30)	2.67 (SD 0.51) 45%	2.67 (SD 0.51) 45%	2.76 (SD 0.56) 46%	2.73 (SD 0.54) 46%	2.73 (SD 0.54) 46%	3.06 (SD 0.60) 51%	NR	NR
Cesarone et al. NRCT study (2019)										
Premenopausal LP vs. BM	LP: 34 BM: 33	NR	*p* < 0.05	*p* < 0.05	*p* < 0.05	*p* < 0.05	*p* < 0.05	*p* < 0.05	NR	NR
Post-menopausal LP vs. BM	LP: 38 BM: 35	NR	*p* < 0.05	*p* < 0.05	*p* < 0.05	*p* < 0.05	*p* < 0.05	*p* < 0.05	NR	NR

Meston et al. (2002) is not included in this table as this study did not measure FSFI. Abbreviations: BM: Best management; FSFI: Female Sexual Function Index; LP: Lady Prelox; NR: Not reported; NRCT: nonrandomized controlled trial; RCT: randomized controlled trial. ^1^ Lubrication reported as frequency of dryness. ^2^ Categories reported as median values. ^3^ Reported as % improved. ^4^ *p*-values were not reported. ^a^ Level of desire. ^b^ Feelings of sexual desire. ^c^ Overall sexual satisfaction. ^d^ Satisfaction with sexual partner.

**Table 3:** Efficacy and Safety Outcomes [10,13,19,20,21,29,30].

	Oxidative Stress ^1^	Menopausal Symptoms Questionnaire ^2,3^	Preclinical Items ^4^	Vaginal Pulse Amplitude ^5^	Subjective Rating of Sexual Arousal ^6^	Hunter’s WHQ	Safety Parameters ^7^
Ito et al. RCT (2001)							
	NR	NR	NR	NR	NR	NR	No AE’s reported
Meston et al. RCT (2002)							
L-Arg + YH 30 min	NR	NR	NR	2.50 ± 3.9	NR	NR	No AE’s reported
L-Arg + YH 60 min	NR	NR	NR	2.66 ± 3.1	NR	NR	No AE’s reported
L-Arg + YH 90 min	NR	NR	NR	2.61 ± 4.3	NR	NR	No AE’s reported
				Placebo (*p* = 0.01), YH (*p* = 0.19)			No AE’s reported
YH 30 min	NR	NR	NR	2.54 ± 2.7	NR	NR	No AE’s reported
YH 60 min	NR	NR	NR	1.56 ± 2.6	NR	NR	No AE’s reported
YH 90 min	NR	NR	NR	2.12 ± 2.6	NR	NR	No AE’s reported
				Placebo (*p* = 0.21)			No AE’s reported
Placebo 30 min	NR	NR	NR	1.70 ± 3.6	NR	NR	No AE’s reported
Placebo 60 min	NR	NR	NR	1.03 ± 2.1	NR	NR	No AE’s reported
Placebo 90 min	NR	NR	NR	2.00 ± 3.5	NR	NR	No AE’s reported
Overall Group Differences	NR	NR	NR	NR	Physical Sexual arousal (*p* = 0.68) Mental sexual arousal (*p* = 0.69) Autonomic Arousal (*p* = 0.82) Positive Affect (*p* = 0.56) Negative affect (*p* = 0.44)	NR	No AE’s reported
Ito et al. RCT (2006)							
	NR	NR	NR	NR	NR	NR	Minor gastric disturbances (*n* = 4) Heavier bleeding during menstruation (*n* = 2) Increased headache (*n* = 1)
Bottari et al. RCT (2012)							
	NR	NR	NR	NR	NR	NR	No AE’s reported
Bottari et al. NRCT (2013)							
LP Baseline	388.29	NR	NR	NR	NR	NR	No AE’s reported
LP 4 weeks	344.28 (*p* < 0.05)	NR	NR	NR	NR	NR	No AE’s reported
LP 8 weeks	332.31 (*p* < 0.05)	NR	NR	NR	NR	NR	No AE’s reported
BM Baseline	389.33	NR	NR	NR	NR	NR	No AE’s reported
BM 4 weeks	377.32 (*p* < 0.05)	NR	NR	NR	NR	NR	No AE’s reported
BM 8 weeks	365.33 (*p* < 0.05)	NR	NR	NR	NR	NR	No AE’s reported
Stanislavov et al. RCT (2014) ^1^							
LP Baseline	NR	Total score different at LP baseline compared to placebo baseline	NR	NR	NR	NR	No AE’s reported
LP 1 month	NR	Total score significantly different (*p* < 0.001) at LP 1 month compared to placebo 1 month; significantly different from baseline	NR	NR	NR	Significantly different (*p* < 0.001) at LP 1 month compared to placebo 1 month in all parameters except attractiveness	No AE’s reported
LP 2 months	NR	Total score significantly different (*p* < 0.001) at LP 2 month compared to placebo 2 month; significantly different from baseline	NR	NR	NR	Significantly different (*p* < 0.001) at LP 1 month compared to placebo 1 month in all parameters	No AE’s reported
Placebo Baseline	NR		NR	NR	NR		No AE’s reported
Placebo 1 month	NR		NR	NR	NR		No AE’s reported
Placebo 2 months	NR		NR	NR	NR		No AE’s reported
Cesarone et al. NRCT study (2019) ^2^							
Premenopausal LP vs. BM	*p* < 0.05 vs. baseline	NR	All improved significantly versus best management (*p* < 0.05)	NR	NR	NR	No AE’s reported
Post-menopausal LP vs. BM	*p* < 0.05 vs. baseline	Significant effect for LP on all symptoms at 8 weeks (*p* < 0.05) ^7^	All improved significantly versus best management (*p* < 0.05)	NR	NR	NR	No AE’s reported

BM: Best Management; L-Arg: l-arginine; LP: Lady Prelox; NR: Not reported; NRCT: nonrandomized controlled trial; RCT: randomized controlled trial; YH: Yohimbine hydrochloride. ^1^ Measure in Carr Units. ^2^ Kupperman’s Index. ^3^ Menopausal Symptoms Score Questionnaire. ^4^ Preclinical Items (vaginal dryness, pain/discomfort during intercourse, mucus, minimal infections, presence of candida, oxidative stress in vaginal mucus, systemic oxidative stress, mild breast tenderness/swelling, hair loss). ^5^ Reported in microvolts (V × 10^−6^). ^6^ Subjective Rating of Sexual Arousal (physical sexual arousal, autonomic arousal, positive affect, negative affect). ^7^ No significant side effects reported unless otherwise noted.

**Table 4:** Risk-of-bias Assessment [10,13,19,20,21,29,30].

	Randomization Process	Confounding Factors	Selection of Participants	Classification of Interventions	Deviations from Intended Interventions	Missing Data	Measurement of Outcomes	Selection of Reported Result	Overall Bias
ROB2 ^1^									
Ito et al. RCT (2001)	Low risk	N/A	N/A	N/A	Low risk	Low risk	Low risk	Low risk	Low risk
Meston et al. RCT (2002)	Low risk	N/A	N/A	N/A	Low risk	Low risk	Low risk	Low risk	Low risk
Ito et al. RCT (2006)	Low risk	N/A	N/A	N/A	Low risk	Low risk	Low risk	Low risk	Low risk
Bottari et al. RCT (2012)	Low risk	N/A	N/A	N/A	Some concerns	Low risk	Low risk	Low risk	Some concerns
Stanislavov et al. RCT (2014)	Some concerns	N/A	N/A	N/A	Low risk	Low risk	Low risk	Low risk	Some concerns
ROBINS-I ^2^									
Bottari et al. NRCT (2013)	N/A	Serious concerns	Low risk	Moderate risk	Low risk	Some concerns	Serious concerns	Low Risk	Serious risk
Cesarone et al. NRCT (2019)	N/A	Serious concerns	Low risk	Moderate risk	Low risk	Low risk	Serious concerns	Low Risk	Serious risk

Abbreviations: NRCT: nonrandomized controlled trial, RCT: randomized controlled trial. ^1^ Confounding factors, Selection of participants and classification of interventions are not included in the ROB2 tool. ^2^ ROBINS-I does not account for randomization as these studies are nonrandomized controlled trials.
